# Total chemical synthesis of ester-linked ubiquitinated proteins unravels their behavior with deubiquitinases†
†Electronic supplementary information (ESI) available. See DOI: 10.1039/c7sc04518b


**DOI:** 10.1039/c7sc04518b

**Published:** 2018-01-11

**Authors:** Hao Sun, Roman Meledin, Sachitanand M. Mali, Ashraf Brik

**Affiliations:** a Schulich Faculty of Chemistry , Technion Israel Institute of Technology , Haifa , 3200008 , Israel . Email: abrik@technion.ac.il

## Abstract

Ester-linked ubiquitinated proteins have been reported by several groups to be involved in ubiquitin signalling. However, due to the lack of the suitable tools to homogeneously produce such conjugates, their exact physiological roles and biochemical behavior remain enigmatic. Here, we report for the first time on the development of a novel synthetic strategy based on total chemical synthesis of proteins to construct ubiquitinated proteins, where ubiquitin is linked to the substrate *via* an ester bond. In this study, we prepared ester- and isopeptide-linked ubiquitinated α-globin and examined their relative behaviors with various deubiquitinases. We found that deubiquitinases are able to cleave the ester linkage with different efficiency relative to the isopeptide-linked substrate. These results may indicate that ester-linked ubiquitinated proteins are natural substrates for deubiquitinases.

## Abstract 2

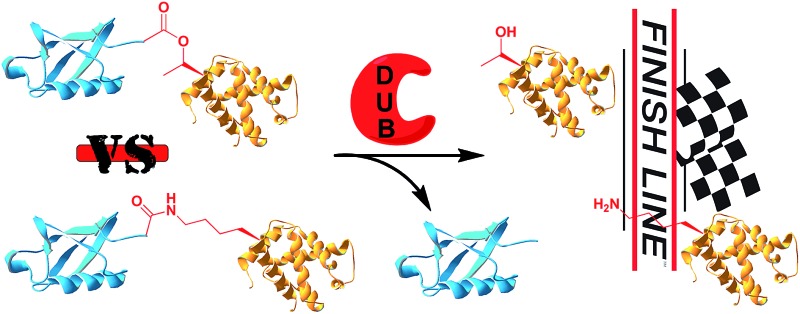
The novel synthetic strategy for preparation of ester linked ubiquitinated proteins was developed. We found that the ester linkage could be cleaved by deubiquitinases with different efficiency relative to the isopeptide-linked substrate.

## Introduction

**Fig. 1:**
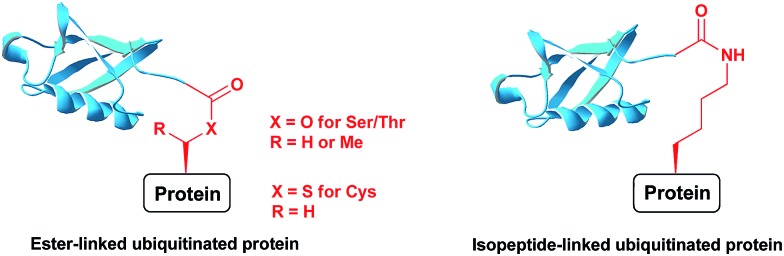
Ubiquitinated proteins with different linkages.

Ubiquitination is a posttranslational modification (PTM) which targets proteins for a variety of processes, such as proteasomal degradation, ER-associated degradation and intracellular trafficking.^1^ The ubiquitin (Ub) monomers form a chain in which its C-terminal Gly can be linked to any of the seven available Lys side chains or the N-terminus of the preceding Ub, yielding a large diversity of polyUb chains.^2^ Ub or any of the polyUb chains can be linked to a protein substrate through a Lys residue of a protein *via* an isopeptide bond. In addition, unusual ester or thioester linkages through Ser/Thr and Cys, respectively, can be formed (Fig. 1).^3^ In these processes, Ub conjugates are assembled by three enzymatic steps including the activating enzyme E1, the conjugating enzyme E2 and the E3 ligase.^4^ Once being installed, the Ub modifier is recognized by Ub binding domains based on location, topology and conformation of the Ub chain^5^ to initiate a specific signal according to the type of ubiquitination. Like many PTMs, ubiquitination is a reversible process where deubiquitinases (DUBs) disassemble the Ub chains or the ubiquitinated proteins, therefore playing crucial roles in Ub signalling.^6^

Despite the fact that most proteins are linked to Ub or polyUb *via* their Lys residue there is growing evidence about the existence and physiological relevance of the “non-canonical” ubiquitination.^7^,^8^ Indeed, it was reported by several groups that proteins lacking Lys or those, where the Lys residues were mutated to Arg can still be ubiquitinated and targeted for the proteasomal or ER-associated degradation.^9^–^11^ For example, the heavy chain of the major histocompatibility complex-I (MHC-I HC) was shown to undergo ubiquitination through Ser, Thr and Lys residues by the viral E3 ligase mK3.^12^ This ubiquitination subsequently stimulates the ER-associated degradation of the MHC-I and thus prevents the immune response against viral infected cells. Notably, in this example and for unclear reason ubiquitination through Ser or Thr residues was found to be preferentially formed than *via* conventional isopeptide linkage. Another important aspect of protein ubiquitination through the Thr/Ser side chains is the nature of the ester linkage. In addition to having a different number of atoms, ester also provides a higher degree of rotational freedom than the corresponding amide bond. These properties could confer unusual surface interactions of the proximal anchored Ub with the Ub binding proteins that might lead to the specific outcomes.^13^ Indeed, Hansen and colleagues reported that the ubiquitination of HC MHC-I *via* its Ser in contrast to Lys leads to subsequent polyUb chain build up, exclusively through the K48 linkage type.^14^

Despite these emerging observations, the field of non-canonical protein ubiquitination is still in its infancy. Here, most reported studies rely heavily on mutagenic approaches, which enable indirect observations of the ubiquitination type. While chemical biologists have already developed powerful toolboxes for the preparation of isopeptide bond based ubiquitinated proteins,^15^–^19^ strategies to assemble ester-linked equivalents are still lacking. Filling this gap would enable the preparation of highly pure and homogenous ester-linked ubiquitinated proteins to investigate the interplay between these conjugates and their modulating enzymes, such as DUBs and E2/E3 ligases, including functional, structural and interactome analyses. In addition, it will increase our knowledge about their exact roles in Ub signalling such as in proteasomal degradation and will enable to explain the differences between canonical *versus* non-canonical types of ubiquitination. Finally, such conjugates could be useful as templates for the development of antibodies, which will open the door for a variety of imaging and diagnostics studies.

In order to prepare desired conjugates by conventional enzymatic approaches, one should identify a specific set of E2/E3 ligases, which in turn have to possess a high selectivity towards the specific location and type of ubiquitination. Since there is a shortage of knowledge about these ligases for the ester-linked ubiquitination, biochemical strategies remain significantly restricted. Chemistry, however, offers an alternative approach for the preparation of various complex conjugates based on the total chemical synthesis of proteins.^20^ Regarding the incorporation of an ester unit in proteins there are only a few examples that were reported. For instance, Dawson and colleagues replaced four backbone amide bonds with the ester in chymotrypsin inhibitor II protein to study its folding and stability.^21^ The Liu group applied the solubilizing tag of polyarginine attached through its C terminus carboxylic acid to the threonine side chain of Autophagosomal Marker Protein LC3-II.^22^ Kent and co-workers prepared proinsulin analogues having an ester linkage between threonine and glutamic acid side chains.^23^ Despite the existence of these examples, there are no reported studies about the chemical preparation of a highly complex ester-linked conjugates such as ubiquitinated proteins. Here, for the first time, we report on the total chemical synthesis of ubiquitinated protein, where ubiquitin is linked to a substrate *via* an ester bond to shed light on the behavior of these conjugates with known DUBs.

## Results and discussion

**Scheme 1:**
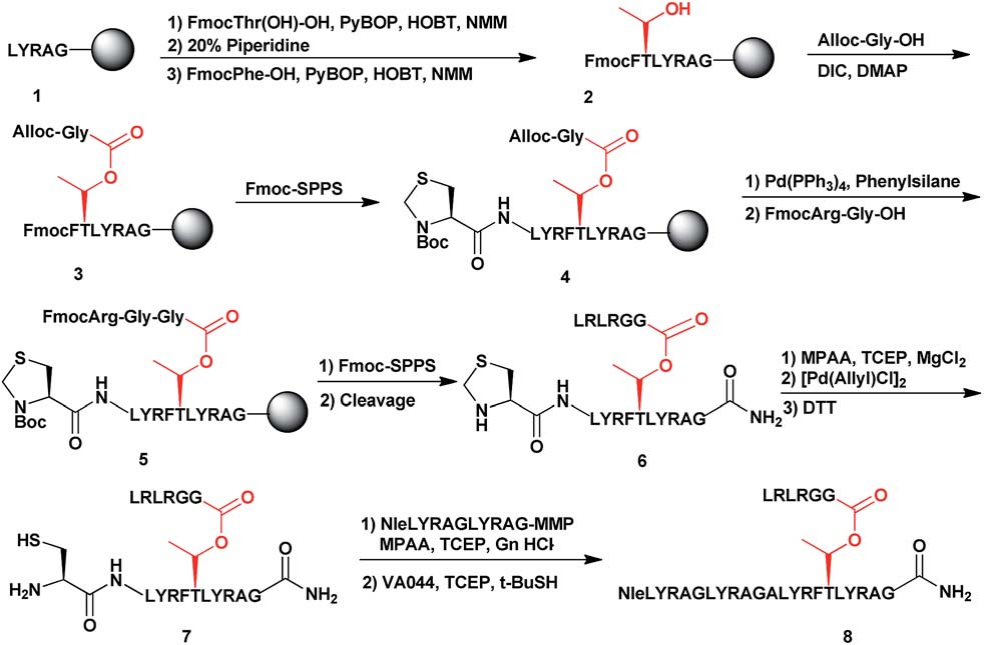
Synthetic strategy for the model peptide containing the ester linkage.

To examine the properties of an ester-linked ubiquitinated protein compared to the isopeptide analogue, we examined first the synthesis of a model peptide containing a branched ester bond (Scheme 1). Such synthesis would enable us to test the stability of the ester bond under different conditions often used in chemical protein synthesis. In this synthesis, the first five amino acids of the 28-mer peptide (**1**) were coupled on the solid support, using standard solid phase peptide synthesis (SPPS).^24^ We then coupled N-terminal Fmoc-Thr-OH with unprotected hydroxyl group using benzotriazol-1-yl-oxytripyrrolidinophosphonium hexafluorophosphate (PyBOP) and hydroxybenzotriazole (HOBT) in presence of *N*-methylmorpholine (NMM).^25^ Next, the N-terminal Fmoc protecting group was removed and phenylalanine was coupled to give **2**. Subsequently, allyloxycarbonyl (Alloc) protected Gly was coupled to the free hydroxyl group of the Thr using *N*,*N*-diisopropylcarbodiimide (DIC) and 4-dimethylaminopyridine (DMAP) to form the ester linkage (**3**). The ester bond could be formed only after the phenylalanine coupling to prevent a five-member ring induced intramolecular attack of the Thr free amine on the ester bond. Next, we completed the synthesis of the backbone peptide and introduced N-terminal thiazolidine (Thz) as the last amino acid to afford **4**. Subsequently, the Alloc protecting group on the branched Gly was removed by Pd(PPh_3_)_4_, followed by coupling the dipeptide Arg–Gly to prevent intramolecular diketopiperazine formation. The branched model sequence was accomplished using standard SPPS to give peptide **6** after the cleavage the deprotection step using TFA. Finally, Thz was deprotected by [Pd(Allyl)Cl]_2_ (ref. 26) to afford peptide **7**, following by native chemical ligation with peptide-thioester^27^ and desulfurization^28^,^29^ to give peptide **8**. Importantly, through all these synthetic steps we did not observe any hydrolysis of the ester linkage (Fig. S1†).

**Scheme 2:**
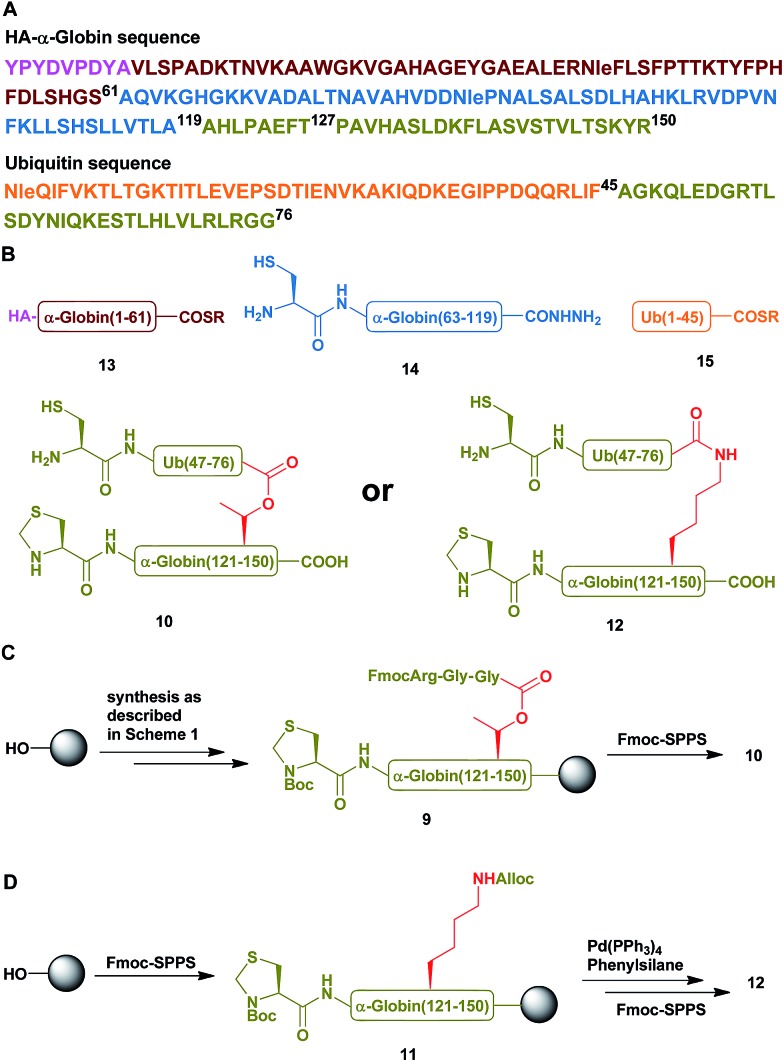
(A) The sequences of HA-α-globin and Ub. (B) The peptide building blocks for the ubiquitinated α-globin analogues. (C & D) Synthetic strategies for the preparation of fragment **10** with the ester linkage and of fragment **12** with the isopeptide linkage, respectively.

**Scheme 3:**
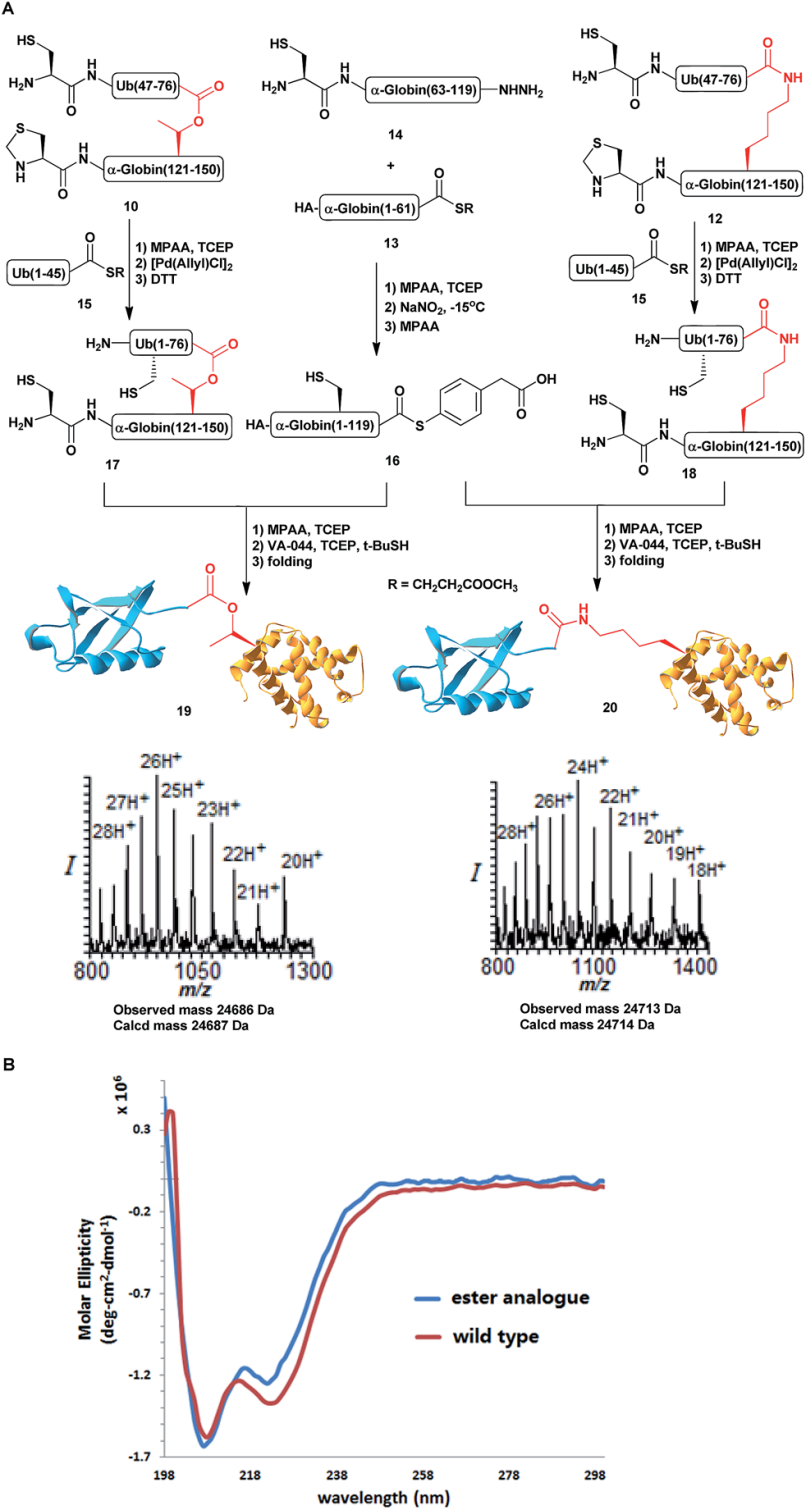
(A) Syntheses of HA-α-globin-Ub having the ester or isopeptide linkages with the observed masses of 24 686 Da (calcd 24 687 Da) and 24 713 Da (calcd 24 714 Da), respectively. (B) CD analysis of the HA-α-globin-Ub analogues.

Having succeeded with the model peptide, we then turned our attention to prepare ester-linked ubiquitinated protein. We chose α-globin as the Ub acceptor protein (Scheme 2A), since in addition to its naturally being ubiquitinated,^30^ it has been also shown to behave as an excellent model for DUB's and proteasomal degradation studies.^31^,^32^ To compare the properties of the ester- and the isopeptide-linked ubiquitinated α-globin, we designed synthetic schemes for both variants. As the anchoring location of Ub we chose Thr127 because of its surface exposure. In addition, α-globin has been reported to undergo ubiquitination at different positions, including the Thr region.^33^ To prepare desired the ester- and isopeptide-linked ubiquitinated HA-α-globin we divided the ester analogue **19** (Scheme 3A) to four fragments: **10**, **13**, **14** and **15** (Scheme 2B), while for the isopeptide analogue **20** (Scheme 3A) we used the four fragments **12**, **13**, **14** and **15** (Scheme 2B). Fragment **10**, Ub(46–76)-α-globin(120–150), which contains an ester linkage *via* the Thr127 side chain was prepared in a similar manner to the model peptide **8** (Scheme 2C & Fig. S2†). On the other hand, fragment **12** bearing the isopeptide bond was synthesized using Alloc protected Lys to enable side chain elongation of the Ub peptide(46–76) at position127 (Scheme 2D & Fig. S3†). This peptide, like **10** has also N-terminal Thz. Next, upon Alloc removal by Pd(PPh_3_)_4_ on resin, SPPS of the Ub sequence was accomplished.^34^ HA-α-globin(1–61)-MMP, **13**, and Ub(1–45)-MMP, **15**, were prepared on the solid support using *N*-acyl-*N*′-methyl-benzimidazolinone (MeNbz) chemistry.^35^ On the other hand, Cys-α-globin(63–119)-NHNH_2_**14** was prepared with *N*-acyl-benzimidazolinone (Nbz) approach.^36^ This was followed by switching **13** and **15** to the thioester precursors and of **14** to hydrazide functionality.^37^ During the cyclization step of fragment **14** in DCM we obtained only ∼30% conversion, while cyclization in DMF according to our previous reports led to a full conversion.^38^ Interestingly, we found that we could obtain the same efficiency of conversion without the need of the extra step of treatment with the base *N*,*N*-diisopropylethylamine.^36^ The N-terminal amino acids in the fragments **10**, **12** and **14** were temporarily changed to Cys to enable subsequent ligation steps, which at later stage will be converted back to Ala *via* desulfurization.

With all fragments in hand, we performed stepwise synthesis of the two variants of HA-α-globin-Ub (Scheme 3A). First, fragment **13** was ligated with fragment **14**, followed by switching of the ligated product to thioester **16**. In parallel, fragment **10** or **12** was ligated with **15**, followed by Thz deprotection to give **17** or **18**, respectively. Finally, fragments **16** was ligated with **17** or **18** and the product was desulfurized in one-pot manner to give the final HA-α-globin-Ub analogue.^39^ The secondary structures of these ubiquitinated proteins were verified by circular dichroism (CD), supporting correct folding (Scheme 3B).

**Fig. 2:**
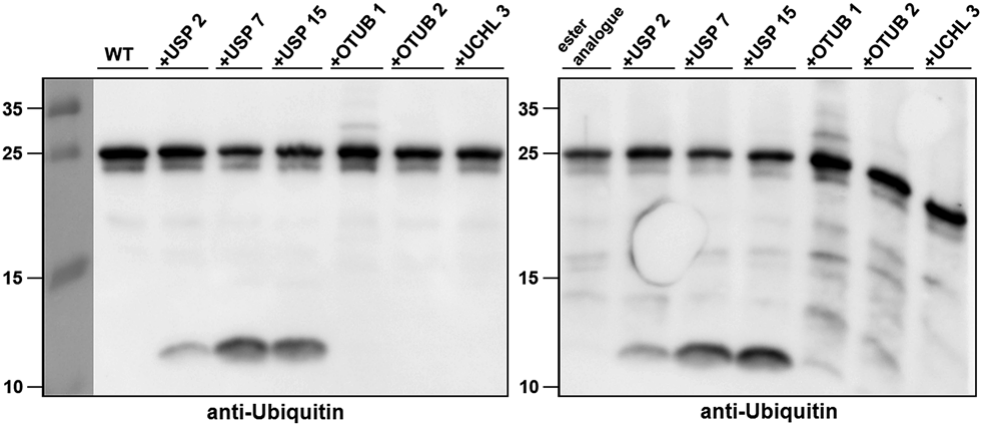
Western blot analysis of ester- and isopeptide-linked HA-α-globin-Ub treated with panel of DUBs. The analogues were treated with 1 : 10 enzyme to substrate molar ratio.

**Fig. 3:**
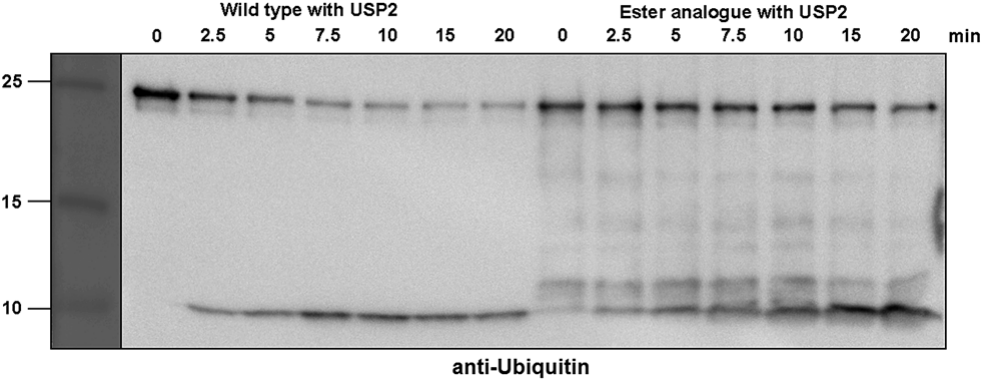
Representative western blot analysis of ester-linked HA-α-globin-Ub (right gel) and wild type (left gel). The analogues were treated with USP2 at 1 : 50 (enzyme : substrate) molar ratio for different time points. Untreated analogues were used as a control.

**Fig. 4:**
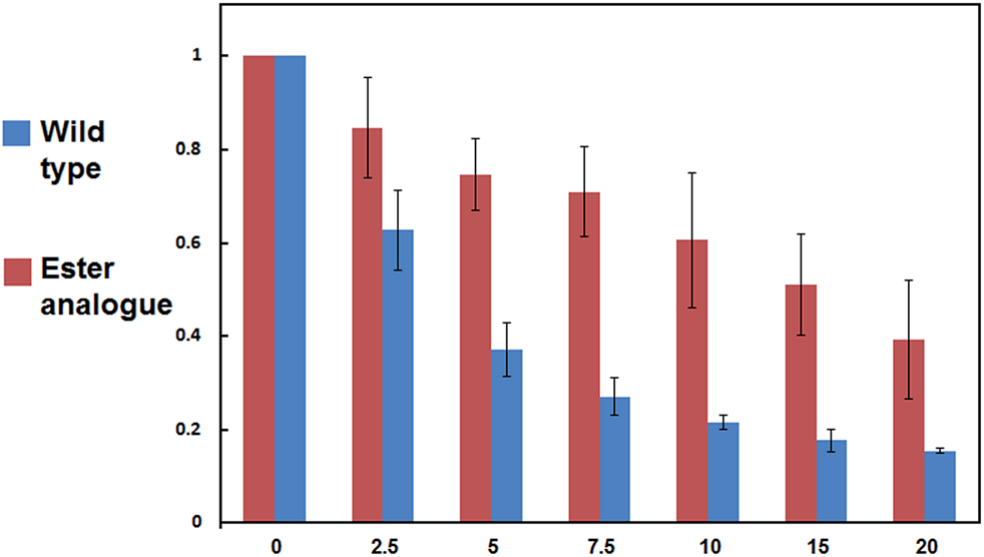
Summary of wild type and ester analogue deubiquitination by USP2, quantified from three independent western blot analyses.

Next, we turned our attention to biochemical characterization of the ester- and isopeptide-linked conjugates using DUBs cleavage assay. The nature of the isopeptide bond is considered chemically more stable than the ester, because of higher stability of the amide's resonance form. However, due to the variation in length, conformation and the structure of the linkages, their recognition by DUBs might vary, which could lead to a difference in cleavage efficiency and DUBs specificities. To examine this, a panel of purified DUBs was incubated with each of the HA-α-globin-Ub variants at 37 °C for 5 minutes, followed by western blot analysis to evaluate the cleavage efficiency (Fig. 2 & S11†). Our results show that USP2, USP7 and USP15 cleave both ester- and isopeptide-linked conjugates with different efficiency. Interestingly, the cleavage of each analogue by USP2 is weaker than by USP7 and USP15. To compare the cleavage efficiency of isopeptide *versus* ester-linked ubiquitinated HA-α-globin we chose to focus on USP2, which was the slowest in the previous experiment. By incubating these analogues with USP2 at different time points we found that the isopeptide-linked HA-α-globin-Ub undergoes faster cleavage than the ester-linked analogue (Fig. 3 & S12†). For example, after 7.5 minutes of incubation, the ester-linked HA-α-globin-Ub exhibited around 30% cleavage, while the isopeptide analogue underwent over 70% (Fig. 4). To examine if this observation depends on the type of DUB we performed similar analysis with USP15, which exhibited with both α-globin-Ub variants faster cleavage efficiencies compared to USP2. Similar to the USP2 case, we found comparable differences in the cleavage of the two analogues, highlighting the effect of the linkage type on the enzymatic cleavage (ESI, Fig. S13†).

These findings are interesting because it shows for the first time the ability of DUBs to cleave an ester bond between Ub and its acceptor protein. Our preliminary analysis shows that the cleavage with two different DUBs is slower compared to the isopeptide bond counterpart. We cannot exclude, however, the possible effect of the natural protein substrate, which dictates conformation and exposure of the ester linkage toward DUBs, on this process. As it is known that proteins labeled for the degradation by Ub, may be rescued by the action of DUBs, it is tempting to assume that difference in the removal of Ub could tune the degradation rate by the proteasome.^32^,^40^ Hence, the more stable Ub-substrate the less chance to be rescued from degradation. In this context, it has been shown that lysine-less mutant of NS-1 immunoglobulin κ LC, where ubiquitination occurs *via* the ester linkage, degrades faster compared to the wild type protein.^41^ Further support for these hypotheses requires detailed studies, including comprehensive kinetic characterizations of their cleavage by DUBs. In addition, the effect of Ub location as well as specific type of ester bond (Ser *vs.* Thr) should also be examined.

## Conclusions

In summary, we have developed for the first time a new synthetic strategy to chemically prepare ubiquitinated proteins where Ub is linked to a protein substrate *via* an ester bond. This allowed us to examine the activity of these types of conjugates with various DUBs. Our study demonstrated that ester-linked ubiquitinated protein could serve as substrates for DUBs, albeit being cleaved at slower rate compared to the isopeptide ubiquitinated protein counterpart. This novel platform opens the door to investigate in more detailed analysis the effect of the ester connectivity on various aspects of Ub signalling and whether such a linkage could be another layer of regulation.

## Conflicts of interest

There are no conflicts to declare.

## Supplementary Material

Supplementary information

## References

[cit1] Glickman M. H., Ciechanover A. (2002). Physiol. Rev..

[cit2] Komander D., Rape M. (2012). Annu. Rev. Biochem..

[cit3] Kravtsova-Ivantsiv Y., Sommer T., Ciechanover A. (2013). Angew. Chem., Int. Ed. Engl..

[cit4] Pickart C. M. (2001). Annu. Rev. Biochem..

[cit5] Dikic I., Wakatsuki S., Walters K. J. (2009). Nat. Rev. Mol. Cell Biol..

[cit6] Reyes-Turcu F. E., Wilkinson K. D. (2009). Chem. Rev..

[cit7] Kravtsova-Ivantsiv Y., Ciechanover A. (2012). J. Cell Sci..

[cit8] McDowell G. S., Philpott A. (2013). Int. J. Biochem. Cell Biol..

[cit9] Cadwell K., Coscoy L. (2005). Science.

[cit10] Ishikura S., Weissman A. M., Bonifacino J. S. (2010). J. Biol. Chem..

[cit11] Vosper J. M., McDowell G. S., Hindley C. J., Fiore-Heriche C. S., Kucerova R., Horan I., Philpott A. (2009). J. Biol. Chem..

[cit12] Wang X., Herr R. A., Chua W. J., Lybarger L., Wiertz E. J., Hansen T. H. (2007). J. Cell Biol..

[cit13] Wang X., Herr R. A., Hansen T. H. (2012). Traffic.

[cit14] Wang X., Herr R. A., Rabelink M., Hoeben R. C., Wiertz E. J., Hansen T. H. (2009). J. Cell Biol..

[cit15] Weller C. E., Pilkerton M. E., Chatterjee C. (2014). Biopolymers.

[cit16] van Tilburg G. B., Elhebieshy A. F., Ovaa H. (2016). Curr. Opin. Struct. Biol..

[cit17] Pham G. H., Strieter E. R. (2015). Curr. Opin. Chem. Biol..

[cit18] Meledin R., Mali S. M., Brik A. (2016). Chem. Rec..

[cit19] Mali S. M., Singh S. K., Eid E., Brik A. (2017). J. Am. Chem. Soc..

[cit20] Bondalapati S., Jbara M., Brik A. (2016). Nat. Chem..

[cit21] Beligere G. S., Dawson P. E. (2000). J. Am. Chem. Soc..

[cit22] Huang Y. C., Li Y. M., Chen Y., Pan M., Li Y. T., Yu L., Guo Q. X., Liu L. (2013). Angew. Chem., Int. Ed. Engl..

[cit23] Sohma Y., Hua Q. X., Whittaker J., Weiss M. A., Kent S. B. (2010). Angew. Chem., Int. Ed. Engl..

[cit24] Merrifield R. B. (1963). J. Am. Chem. Soc..

[cit25] Fischer P. M., Retson K. V., Tyler M. I., Howden M. E. (1991). Int. J. Pept. Protein Res..

[cit26] Jbara M., Maity S. K., Seenaiah M., Brik A. (2016). J. Am. Chem. Soc..

[cit27] Dawson P. E., Muir T. W., Clark-Lewis I., Kent S. B. (1994). Science.

[cit28] Wan Q., Danishefsky S. J. (2007). Angew. Chem., Int. Ed. Engl..

[cit29] Yan L. Z., Dawson P. E. (2001). J. Am. Chem. Soc..

[cit30] Shaeffer J. R. (1983). J. Biol. Chem..

[cit31] Hemantha H. P., Bavikar S. N., Herman-Bachinsky Y., Haj-Yahya N., Bondalapati S., Ciechanover A., Brik A. (2014). J. Am. Chem. Soc..

[cit32] Singh S. K., Sahu I., Mali S. M., Hemantha H. P., Kleifeld O., Glickman M. H., Brik A. (2016). J. Am. Chem. Soc..

[cit33] Shaeffer J. R. (1994). J. Biol. Chem..

[cit34] Kumar K. S., Spasser L., Ohayon S., Erlich L. A., Brik A. (2011). Bioconjugate Chem..

[cit35] Blanco-Canosa J. B., Nardone B., Albericio F., Dawson P. E. (2015). J. Am. Chem. Soc..

[cit36] Blanco-Canosa J. B., Dawson P. E. (2008). Angew. Chem., Int. Ed. Engl..

[cit37] Fang G. M., Li Y. M., Shen F., Huang Y. C., Li J. B., Lin Y., Cui H. K., Liu L. (2011). Angew. Chem., Int. Ed. Engl..

[cit38] Siman P., Blatt O., Moyal T., Danieli T., Lebendiker M., Lashuel H. A., Friedler A., Brik A. (2011). ChemBioChem.

[cit39] Moyal T., Hemantha H. P., Siman P., Refua M., Brik A. (2013). Chem. Sci..

[cit40] Komander D., Clague M. J., Urbe S. (2009). Nat. Rev. Mol. Cell Biol..

[cit41] Shimizu Y., Okuda-Shimizu Y., Hendershot L. M. (2010). Mol. Cell.

