# Supplementary material for: Total chemical synthesis of ester-linked ubiquitinated proteins unravels their behavior with deubiquitinases
Source: Chem Sci. 2018 Jan 11;9(6):1661–5. doi: 10.1039/c7sc04518b (PMC5887810; doi:10.1039/c7sc04518b)
Supplement: Supplementary file 1 [file SC-009-C7SC04518B-s001.pdf]

Electronic Supplementary Material (ESI) for Chemical Science.
This journal is © The Royal Society of Chemistry 2017

Supporting information

Total Chemical Synthesis of Ester-Linked Ubiquitinated Proteins Unravels their Behavior with Deubiquitinases

Hao Sun, Roman Meledin, Sachitanand M. Mali and Ashraf Brik*

Schulich Faculty of Chemistry, Technion-Israel Institute of Technology, Haifa 3200008, Israel.

Corresponding author: abrik@technion.ac.il

Contents

General methods S3

Synthesis of the dipeptide Fmoc-Arg(Pbf)-Gly-OH..........S6

Synthesis of the model peptide containing ester linkage..........S7

Synthesis of Cys-Ub(47-76) ester-linked Thz-α-globin(121-150) S10

Synthesis of Cys-Ub(47-76) isopeptide-linked Thz-α-globin(121-150) S12

Synthesis of HA-α-globin(1-61)-MMP S13

Synthesis of Cys-α-globin(63-119)-$NHNH_2$ S15

Synthesis of HA-α-globin(1-119)-MPAA S17

Synthesis of Ub ester-linked Cys-α-globin(121-150)..........S19

Synthesis of Ub ester-linked HA-α-globin S21

Synthesis of Ub isopeptide-linked Cys-α-globin(121-150)..........S23

Synthesis of Ub isopeptide-linked HA-α-globin S25

Deubiquitination assays of Ub ester-linked HA-α-globin and wild type with different DUBs..........S27

Deubiquitination assays of Ub ester-linked HA-α-globin and wild type with USP2 and USP15 ....S28

References..........S29

General methods

Solid Phase Peptide Synthesis (SPPS) was carried out manually in syringes, equipped with Teflon filters, purchased from Torviq or by using an automated peptide synthesizer (CS336X, CSBIO). Analytical grade dimethylformamide (DMF) was purchased from Biotech. Commercial reagents were used without further purification. Resins were purchased from Creosalus and all protected amino acids were purchased from GL Biochem. The activating reagents N,N,N′,N′-Tetramethyl-O-(1H-benzotriazol-1-yl)uroniumhexafluorophosphate (HBTU), 1-Hydroxybenzotriazolemonohydrate (HOBt), O-(1H-6-Chlorobenzotriazole-1-yl)-1,1,3,3-tetramethyluronium hexafluorophosphate (HCTU), 1-[Bis(dimethylamino) methylene]-1H-1,2,3-triazolo[4,5-b]pyridinium 3-oxidhexafluorophosphate (HATU) were purchased from Luxembourg Bio Technologies. Unless otherwise mentioned, all reactions were carried out at room temperature.

List of the protected amino acids used in peptides synthesis: Fmoc-Gly-OH, Fmoc-Ala-OH, Fmoc-Val-OH, Fmoc-Leu-OH, Fmoc-Ile-OH, Fmoc-Phe-OH, Fmoc-Pro-OH, Fmoc-His(Trt)-OH, Fmoc-Asn(Trt)-OH, Fmoc-Gln(Trt)-OH, Fmoc-Arg(Pbf)-OH, Fmoc-Lys(Boc)-OH, Fmoc-Tyr(^{t}Bu)-OH, Fmoc-Ser(^{t}Bu)-OH, Fmoc-Thr(^{t}Bu)-OH, Fmoc-Asp(O^{t}Bu)-OH, Fmoc-Glu(O^{t}Bu)-OH, Fmoc-Nle-OH, Fmoc-Trp(Boc)-OH, Fmoc-Leu-Ser(ψMe,MePro)-OH, Fmoc-Ile-Thr(ψMe,MePro)-OH, Fmoc-Asp(OtBu)-(Dmb)Gly-OH, Fmoc-Lys(Boc)-Thr(ψMe,MePro)-OH, Fmoc-Val-Thr(ψMe,MePro)-OH, Fmoc-Ser(tBu)-Thr(ψMe,MePro)-OH, Fmoc-Leu-Thr(ψMe,MePro)-OH, Boc-Cys(Trt)-OH, Boc-Cys(Thz)-OH, Boc-Nle-OH

3-Amino-4-methylamino-benzoic acid (Fmoc-MeDbz),[1] 3,4-diamino-benzoic acid (Fmoc-Dbz),[2] Alloc-Gly-OH were prepared using the reported procedure.[3]

Ub(1-45)-MMP was synthesized based on the strategy reported previously,[4] but using *N*-acyl-*N*-methylacylurea (MeNbz) chemistry for the C-terminal.

HPLC for peptide analysis and purification

Analytical HPLC was performed on a Thermo instrument (Dionex Ultimate 3000) using analytical columns Xbridge (Waters, BEH300 C4, 3.5μm, 4.6 × 150 mm) and XSelect (Waters, CSH C18, 3.5 μm, 4.6 × 150 mm) at a flow rate of 1.2 mL/min. Preparative HPLC was performed on a Thermo instrument (Dionex Ultimate 3000) using Phenomenex Jupiter, 10μm, C18/C4 300 Å, 250 × 22.4 mm and Waters XSelect, C4, 10 μm, 250 × 19 mm at a flow rate of 15 mL/min. Semi preparative HPLC was performed on a Thermo instrument (Dionex Ultimate 3000) using Phenomenex Jupiter C4 10 μm, 300 Å, 250 × 10 mm column, at a flow rate of 4 mL/min. All synthetic products were purified by HPLC and characterized by mass spectrometry using LCQ Fleet Ion Trap (Thermo Scientific). All calculated masses have been reported as an average isotope composition.

Buffer A: 0.1% TFA in water; buffer B: 0.1% TFA in acetonitrile.

Fmoc-SPPS: Fmoc-SPPS was carried on automated peptide synthesizer in presence of 4 equiv of AA, 4 equiv of HCTU, and 8 equiv of DIEA to the initial loading of the resin for 50 mins. The dipeptides were coupled manually using 2 equiv of AA, 2 equiv of HATU, and 4equiv of DIEA to the initial loading of the resin for 2 h.

Cleavage of the peptide from resin: To 0.05 mmol dried peptide-resin, a mixture of trifluoroacetic acid (TFA): triisopropylsilane (TIS): dithiothreitol (DTT): water (H_2O) (90:2.5:2.5:5) (5 ml) was added, and the reaction mixture was shaken for 2 h. The resin was removed by filtration and the filtrate was added dropwise to 30 mL of cold ether. The resin was washed with additional 1 mL TFA (two cycles). To precipitate the peptide, the combined filtrate with ether was centrifuged. The precipitate was dissolved in acetonitrile-water for freeze drying in the lyophilizer to give the crude peptide.

Circular Dichroism Analysis: CD spectra were recorded in a Chirascan (Applied Photophysics) instrument. The samples were first dissolved in 6M Gn·HCl buffer (5%

of the total volume) and then diluted with TRIS buffer (20 mM Tris base, 100 mM NaCl, pH 7.3).

Antibodies: Polyclonal rabbit anti-ubiquitin antibody was purchased from Boston BioChem. Secondary Goat pAb to Rb HRP IgG (HRP) conjugate was purchased from Biorad.

Synthesis of the dipeptide Fmoc-Arg(Pbf)-Gly-OH

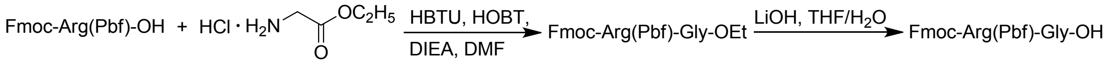

21 22 23 24

To the mixture of Fmoc-Arg(Pbf)-OH **21** (10 mmol, 6.48 g), HBTU (15 mmol, 5.69 g) and HOBT (15 mmol, 2.3 g) dissolved in 50 mL DMF, DIEA (20 mmol, 3.5 mL) was added. After the mixture was stirred for 5 min at room temperature, glycine ethyl ester hydrochloride **22** (20 mmol, 2.8 g) was added. The reaction was stirred for another 1 h. The progress of the reaction was monitored by TLC. Then the reaction was quenched by adding 30 mL water and extracted with EtOAc. The combined organic phase was washed with brine, dried over Na_2SO_4, concentrated and purified using flash column chromatography (ethyl acetate/n-hexane/DIEA = 90/10/0.5) to give the dipeptide ethyl ester **23** (6.6 g, 86%).

To the solution of the dipeptide ethyl ester **23** (6.6 g) in THF-water (4:1) (100 mL) at 0 ºC, the solution of $LiOH·H_2O$ (793 mg, 19mmol) in 20 mL water was added in small portions over a period of 10 mins. After stirring at 0 ºC for another 40 mins, the reaction mixture was adjusted to pH 3-4 by adding 2.5% (w/v) citric acid solution (3.96 g citric acid monohydrate dissolving in 158 mL water), and extracted using ethyl acetate. The combined organic layers was washed with brine, dried over Na_2SO_4, concentrated, and purified using flash column chromatography ($MeOH/CHCl_3$ = 15/85) to give final product **24** as white solid (4.5 g, 71%) (observed mass 706.3, calcd 705.8).

Synthesis of the model peptide containing ester linkage

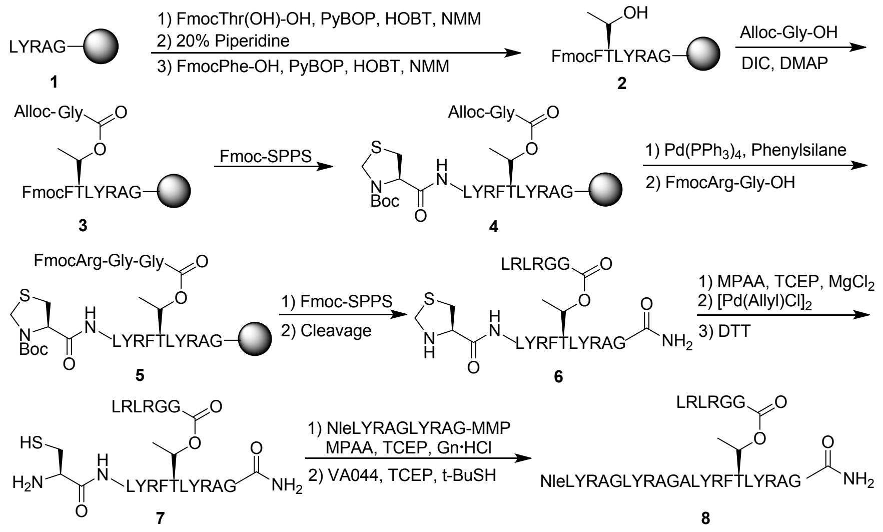

SPPS: The synthesis of peptide **1** was carried out on a Rink amide resin (0.1 mmol scale) followed the standard Fmoc-SPPS protocol. Fmoc-Thr-OH and Fmoc-Phe-OH were coupled manually using 4 equiv of AA, 4 equiv of benzotriazol-1-yl-oxytripyrrolidinophosphoniumhexafluorophosphate (PyBOP) and HOBt, 8 equiv of *N*-methylmorpholine in DMF for 1h to afford **2**. Then Alloc-Gly-OH was coupled to the unprotected Thr with 10 equiv of *N,N'*-diisopropylcarbodiimide (DIC), 2 equiv of 4-dimethylaminopyridine (DMAP) in DMF for 2 h to give **3**. The next amino acids were coupled by standard Fmoc-SPPS to give **4**. After the protectiong group Alloc on Gly was removed by treating the 0.1 mmol resin with a mixture of $Pd(PPh_3)_4$ (40 mg, 0.035 mmol) and phenylsilane (247 µL, 2 mmol) in 2 mL of dry DCM for 1 h, the dipeptide Fmoc-Arg(Pbf)-Gly-OH was coupled manually with 2 equiv of HATU and 4 equiv of DIEA in DMF to give **5**. The remaining amino acids were coupled by standard Fmoc-SPPS to give the crude peptide **6**. The progress of SPPS was monitored by analytical HPLC using C18 column with a gradient of 0-60%B over 30 min.

Thz removal[5, 6]: Crude peptide **6** (5 mg, 2.47×10^{-3} mmol**)** was dissolved in 6 M Gn·HCl, 200 mM phosphate buffer (1235 µL) containing 4-mercaptophenylacetic acid (MPAA) (20 mM) and tris(2-carboxyethyl)phosphine hydrochloride (TCEP) (10

mM) at pH 7.0. Then 50 equiv of $MgCl_2$ (11.7 mg, 0.12 mmol) was added to the reaction mixture and the reaction was incubated at 37 ºC for 10 min. Then the reaction mixture was treated with 15 equiv of $[Pd(Allyl)Cl]_2$ (13.5 mg, 3.7×10^{-2} mmol) and the reaction was incubated at 37 ºC for 1 h for a complete Thz removal. The reaction was quenched by adding 60 equiv of DTT, followed by centrifugation and the supernatant was injected into the HPLC to monitor the progress of the reaction using C18 column with a gradient of 0-60%B over 30 min. Peptide **7** was purified using a C4 semi-preparative column and with the same gradient of the solvent system (3.1 mg, 62 % yield).

Ligation and desulfurization: Peptide **7** (1.0 mg, 5.0×10^{-4} mmol) and NleLYRAGLYRAG-MMP (0.81 mg, 6×10^{-4} mmol) were dissolved in 6 M Gn·HCl, 200 mM phosphate buffer (250 μL) containing MPAA (100 mM) and TCEP (50 mM) at pH 7.2. The reaction was incubated at 37 ºC for 3 h for completion. The reaction mixture was purified using a C4 semi-preparative column with the gradient of 0-60% B over 30 min solvent system to obtain the ligation product. Then the dried ligation product was dissolved in 6 M Gn·HCl (200 uL) and subjected for the desulfurization by treating with TCEP (16 mg, 0.25 mM), VA-044 (4.0 mg, 50 equiv) and t-BuSH (22 μL) at 37 ºC for 6 h. The progress of the reaction was monitored using analytical HPLC (C18 column) with the gradient of 0-60% B over 30 min. The final peptide **8** was purified using a C4 semi-preparative column with the gradient of 0-60% B over 30 min solvent system to obtain (0.8 mg, 50 % yield).

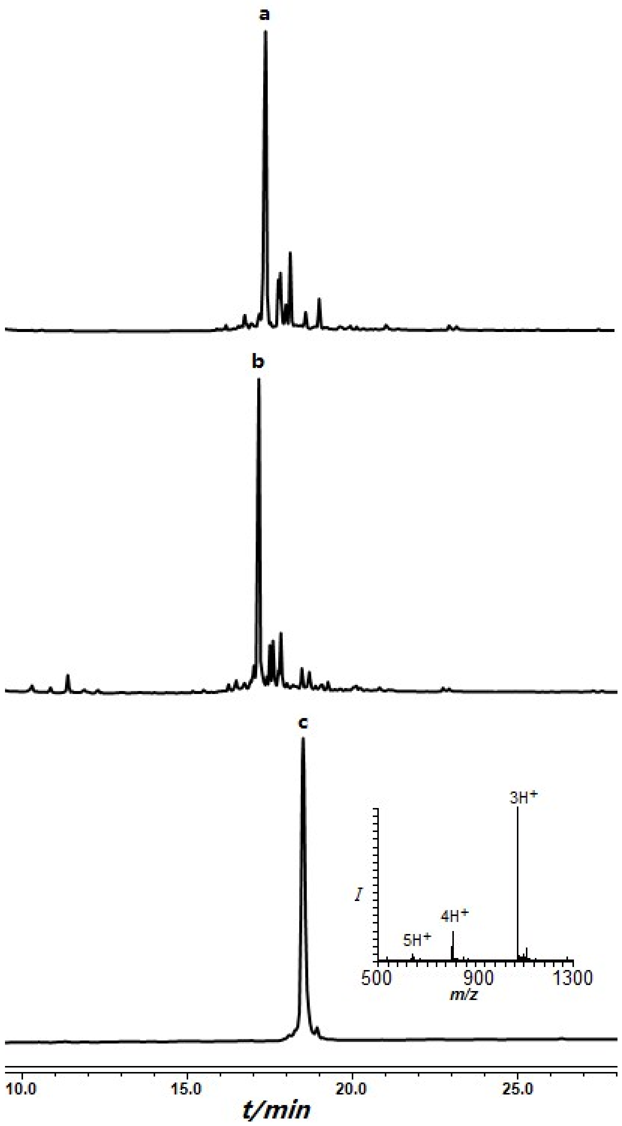

Figure S1. Synthesis of the model peptide: A) Analytical HPLC of the crude peptide **6** with the ester linkage (peak **a**, observed mass 2026 Da, calcd 2026.4 Da). B) HPLC of the deprotection of Thz by Pd after 30 mins, peak **b** corresponds to peptide **7** (observed mass 2014 Da, calcd. 2014.4 Da). C) Analytical HPLC and mass of purified peptide **8** after ligation and desulfurization (peak **c**, observed mass 3217 Da, calcd 3216.8 Da).

Synthesis of Cys-Ub(47-76) ester-linked Thz-α-globin(121-150)

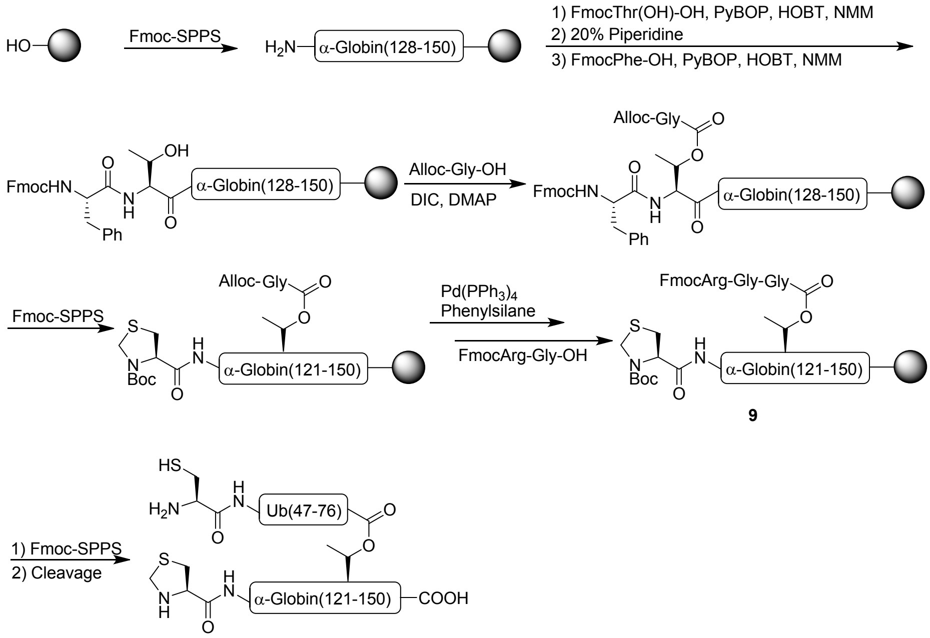

Peptide **10** was prepared according to the synthesis of model peptide **6** described above. The first amino acid Fmoc-Arg(Pbf)-OH was coupled with 10 equiv of HCTU, 20 equiv of DIEA on 0.2 mmol Wang resin for 1h (X2), followed by standard Fmoc-SPPS to couple the next 21 amino acids of α-globin. Fmoc-Thr(OH)-OH and Fmoc-Phe-OH were coupled manually using 4 equiv of AA, 4 equiv of PyBOP and HOBt, 8 equiv of NMM in DMF for 1h. Alloc-Gly-OH was coupled to the unprotected Thr127 with 10 equiv of DIC, 2 equiv of DMAP in DMF for 2 h. The next eight amino acids of the globin sequence were coupled by standard Fomc-SPPS. The Alloc protecting group was then removed by treating the 0.2 mmol resin with a mixture of $Pd(PPh_3)_4$ (81 mg, 0.07mmol) and phenylsilane (494 μL, 4 mmol) in 4 mL of dry DCM for 1 h. The dipeptide Fmoc-Arg(Pbf)-Gly-OH was coupled by manually with 2 equiv of HATU and 4 equiv of DIEA in DMF to afford **9**. The remaining amino acids were coupled by standard Fmoc-SPPS to give the final peptide **10**. The SPPS was monitored by analytical HPLC using C4 column with a gradient of 0-60%B over 30 min. The peptide was cleaved from the resin and purified using a C18 preparative column and with the same gradient of the solvent system to obtain **10** (69 mg, 5% yield).

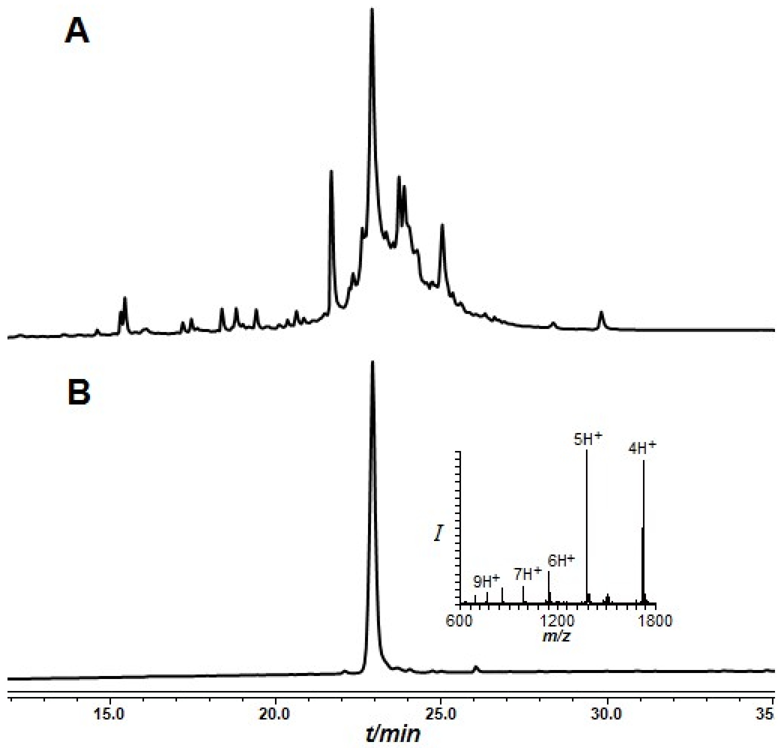

Figure S2. Synthesis of fragment **10** Cys-Ub(47-76) ester-linked Thz-α-globin(121-150): A) Analytical HPLC of crude fragment **10**. B) Analytical HPLC and mass analysis of purified fragment **10** (observed mass 6884 Da, calcd 6884.9 Da).

Synthesis of Cys-Ub(47-76) isopeptide-linked Thz-α-globin(121-150)

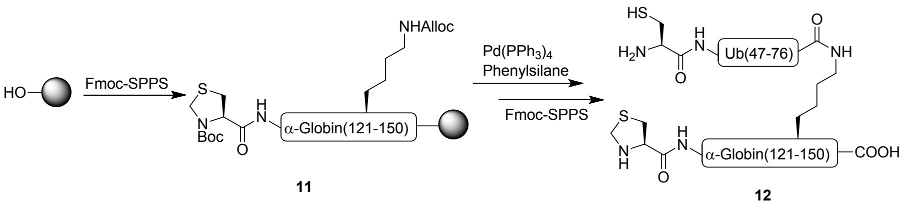

Fmoc-Arg(Pbf)-OH was coupled with 10 equiv of HCTU, 20 equiv of DIEA on 0.2 mmol Wang resin for 1h (X2), followed by standard Fmoc-SPPS to couple the remaining amino acids to give **11**. Thr127 position on HA-α-globin was mutated to Lys. After Alloc protecting group on Lys127 was deprotected by treating the 0.2 mmol resin with a mixture of $Pd(PPh_3)_4$ (81 mg, 0.07mmol) and phenylsilane (494 µL, 4 mmol) in 2 mL of dry DCM for 1 h, followed Gly coupling (two cycles). The remaining amino acids were coupled by standard Fmoc-SPPS to complete the peptide synthesis. The progress was monitored by analytical HPLC using C4 column with a gradient of 0-60%B over 30 min. Finally, the peptide was cleaved from the resin and purified using a C18 preparative column with the same gradient of the solvent system to obtain **12** (97 mg, 7 % yield).

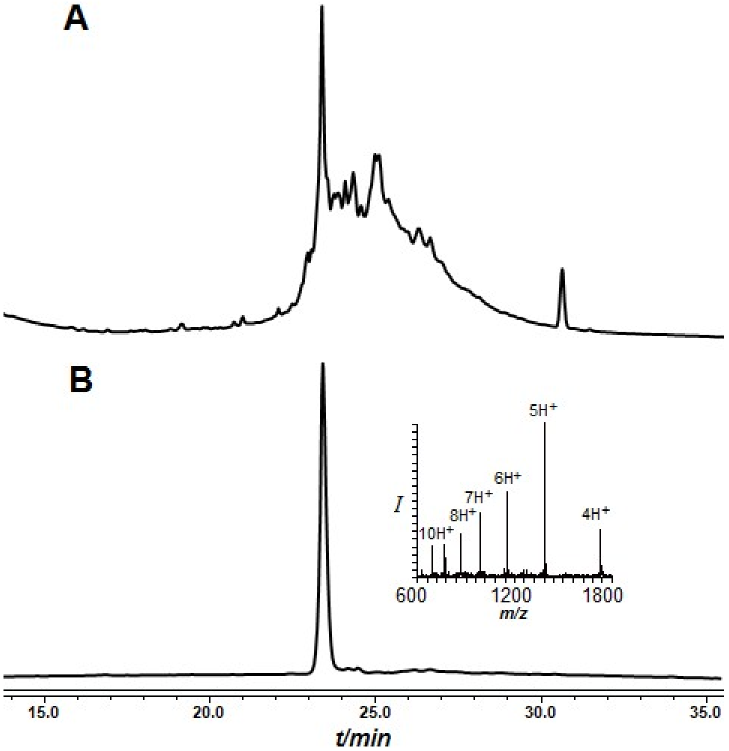

Figure S3. Synthesis of fragment **12** Cys-Ub(47-76) isopeptide-linked Thz-α-globin(121-150): A) Analytical HPLC of the crude fragment. B) Analytical HPLC and mass analysis of purified fragment

12 (observed mass 6912 Da, calcd 6912.0 Da).

Synthesis of HA-α-globin(1-61)-MMP

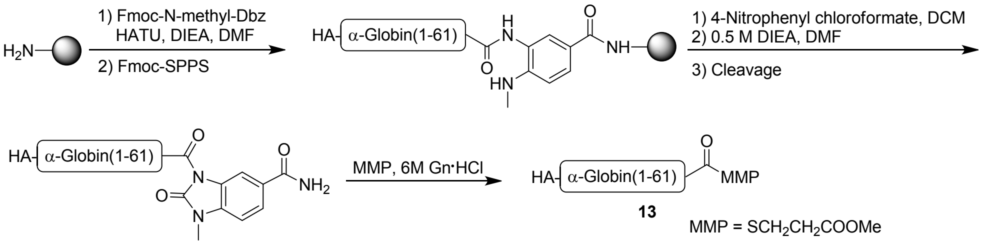

The swelled Fmoc Rink amide resin (0.1 mmol) was deprotected first and coupled with 4 equiv of Fmoc-MeDbz, 4 equiv of HATU and 8 equiv of DIEA for 2 h. The first amino acid Ser was coupled with 4 equiv of HCTU and 8 equiv of DIEA for 45 min (X2), followed by Fmoc-SPPS to couple the remaining amino acids. After completion of the synthesis, the peptide-resin was dried and treated with a solution of 4-nitrophenylchloroformate (100 mg, 5 equiv) in 4 mL of dry DCM for 30 min, followed by washing with dry DCM (X2). After the resin was washed with DCM and DMF, it was treated in 4 mL of 0.5 M DIEA in DMF for 10 min (X2). Finally, the peptide-resin was washed with DMF, dried and cleaved to give the crude peptide. In each 2 mL Eppendorf tube, 25 mg of crude peptide was dissolved in 1.8 mL 6 M Gn·HCl, 200 mM phosphate buffer containing methyl 3-mercaptopropionate (MMP) (200 equiv, 80 μL) and the reaction mixture was incubated at 37 ºC for 1 h. The progress of the reaction was monitored using analytical HPLC (C4 column) with the gradient of 0-60% B over 30 min. The final peptide thioester **13** was purified using a C4 preparative column with the same gradient of the solvent system to obtain (89 mg, 13 % yield).

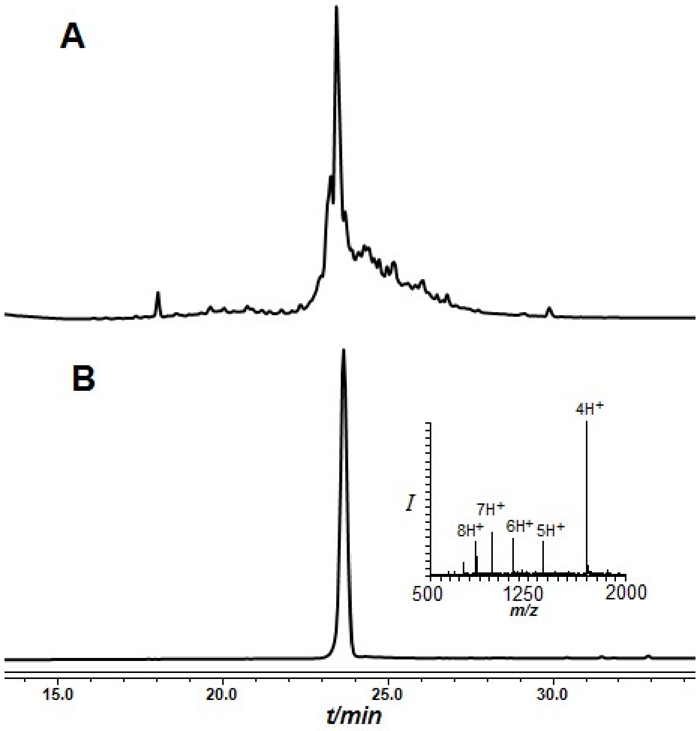

Figure S4. Synthesis of HA-α-globin(1-61)-MMP, fragment **13**: A) Analytical HPLC of crude HA- α-globin(1-61)-MeDbz. B) Analytical HPLC and mass analysis of purified HA-α-globin(1-61)-MMP **13** (observed mass 6087 Da, calcd 6807.4 Da).

Synthesis of Cys-α-globin(63-119)-$NHNH_2$

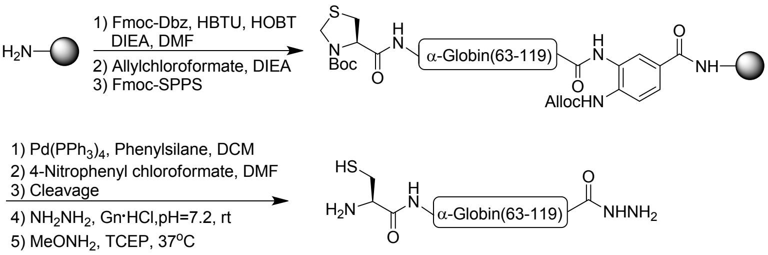

14

The swelled Fmoc Rink amide resin (0.1 mmol) was deprotected first and coupled with 4 equiv of Fmoc-Dbz, 4 equiv of HBTU, 4 equiv of HOBT, and 8 equiv of DIEA for 2 h. The resin was treated in allyl chloroformate (1.5 mmol, 159 uL) and DIEA (0.2 mmol, 35 uL) in 2 mL DCM for 10 h to protect the amine from Fmoc-Dbz. The first amino acid Ala was coupled with 4 equiv of HATU and 8 equiv of DIEA for 45 min (X2), followed by Fmoc-SPPS to couple the remaining amino acids. After completion of SPPS, the peptide was treated with a solution of *p*-nitrophenyl chloroformate (100 mg, 5 equiv) in 4 mL DMF for 30 min and washed with DMF (3 × 5mL). This step was repeated two more times. After peptide cleavage from resin and drying, in each 2 mL Eppendorf tube, 25 mg of crude peptide was dissolved in 1.6 mL 6 M Gn·HCl buffer containing hydrazine monohydrate (150 equiv, 29 uL) at pH 7.2. The reaction mixture was kept at room temperature for 1 h, then TCEP (22 mg, 20 equiv) was added and $MeONH_2$·HCl (16 mg, 50 equiv), followed the incubation at 37 ºC for 3 h to unmask the Thz. The progress of the reaction was followed using analytical HPLC (C4 column) with the gradient of 0-60% B over 30 min. Peptide **14** was purified using a C4 preparative column with the same gradient of the solvent system to obtain (74 mg, 12% yield).

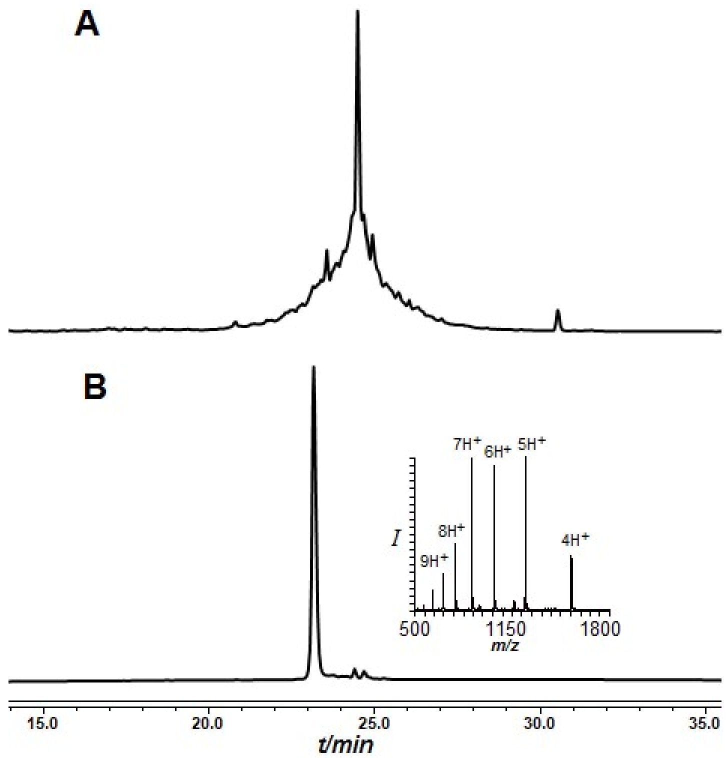

Figure S5. Synthesis of Cys-α-globin(63-119)-$NHNH_2$, fragment **14**: A) Analytical HPLC of crude Thz-α-globin(63-119)-N-Alloc-Dbz. B) Analytical HPLC and mass analysis of purified Cys-α-globin(63-119)-$NHNH_2$ **14** after switching C-terminal to hydrazine and deprotecting Thz group on N-terminal Cys (observed mass 6177 Da, calcd 6177.1 Da).

Synthesis of HA-α-globin(1-119)-MPAA

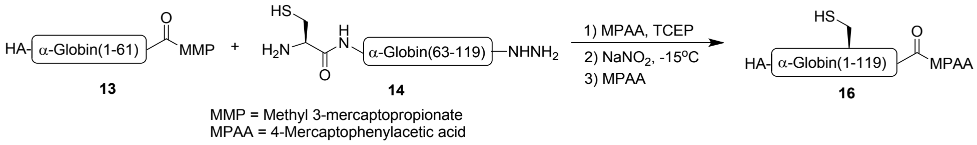

HA-α-globin(1-61)-MMP **13** (15.0 mg, 2.2×10^{-3} mmol) and Cys-α-globin(63-119)-$NHNH_2$ **14** (13.6 mg, 2.2×10^{-3} mmol) were dissolved in 6 M Gn·HCl, 200 mM phosphate buffer (1100 μL) containing MPAA (100 mM) and TCEP (50 mM) at pH 6.8. The reaction was incubated at 37 ºC for 4 h, followed the dialysis in a Slide-A-Lyzer 3.5K dialysis cassette (Thermo scientific, 3.0 mL) in 6 M Gn·HCl, 200 mM phosphate buffer for 12 h. The reaction mixture was adjusted to pH 3 and cooled to -15 ºC with salt ice bath for 20 min. Then 15 equiv of $NaNO_2$ dissolved 10 uL H_2O was added. The reaction mixture was kept under -15 ºC for 20 min. Subsequently, 30 equiv of MPAA dissolving in 6 M Gn·HCl buffer (adjust to pH 6.0) was added to the mixture, followed by adjusting to pH 6.0. After the reaction mixture was kept at room temperature for 7 min and was added the TCEP (63 mg, 0.22 mmol), it was injected to preparative HPLC for purification.[7] The progress of the reaction was followed using analytical HPLC (C4 column) with the gradient of 0-60% B over 30 mins. The product **16** was purified using a C4 preparative column with the gradient of 5-55% B over 45 min solvent system to obtain in 36% yield (10.2 mg) for the three-steps.

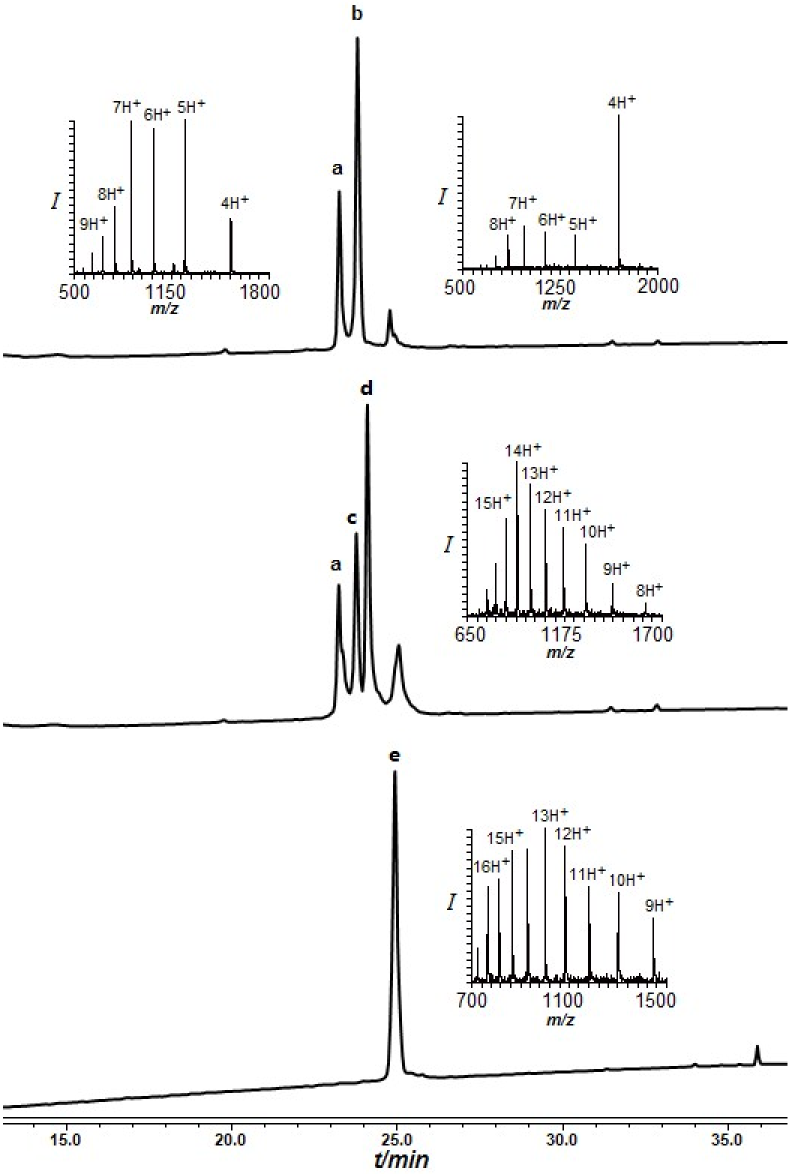

Figure S6. Synthesis of HA-α-globin(1-119)-MPAA **16**: A) Analytical HPLC and mass traces of the ligation at 0 min, peak **a** corresponds to Cys-α-globin(63-119)-$NHNH_2$ **14** and peak **b** corresponds to HA-α-globin(1-61)-MMP **13**. B) Ligation after 1 h, peak **c** corresponds to HA-α-globin(1-61)-MPAA, peak **d** corresponds the ligation product with observed mass 12863 Da (calcd 12864.6 Da). C) Analytical HPLC of the purified **16** after switching to MPAA (peak **e**, observed mass 12999 Da, calcd 13000.6 Da).

Synthesis of Ub ester-linked Cys-α-globin(121-150)

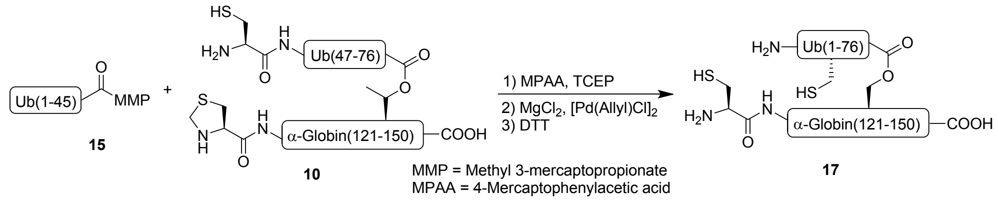

Cys-Ub(47-76) ester-linked Thz-α-globin(121-150) **10** (13.8 mg, 2.0×10^{-3} mmol) and Ub(1-45)-MMP **15** (12.5 mg, 2.4×10^{-3} mmol) were dissolved in 6 M Gn·HCl, 200 mM phosphate buffer (1000 μL) containing MPAA (60 mM) and TCEP (30 mM) at pH 7.2. The reaction was incubated at 37 ºC for 4 h. Then 50 equiv $MgCl_2$ (9.5 mg) was added to the reaction mixture and the reaction was incubated at 37 ºC for 10 min. After that, the reaction mixture was treated with 15 equiv of $[Pd(Allyl)Cl]_2$ (11 mg) and incubated at 37 ºC for 1 h to complete the Thz removal. The reaction was quenched by adding 60 equiv of DTT, followed by centrifugation and the supernatant was injected into the HPLC for analysis and purification. The progress of the reaction was monitored using analytical HPLC (C4 column) with the gradient of 0-60% B over 30 min. The product **17** was purified using a C4 preparative column with the gradient of 5-55% B over 45 min solvent system to obtain to 9.8 mg from the product (41 % yield).

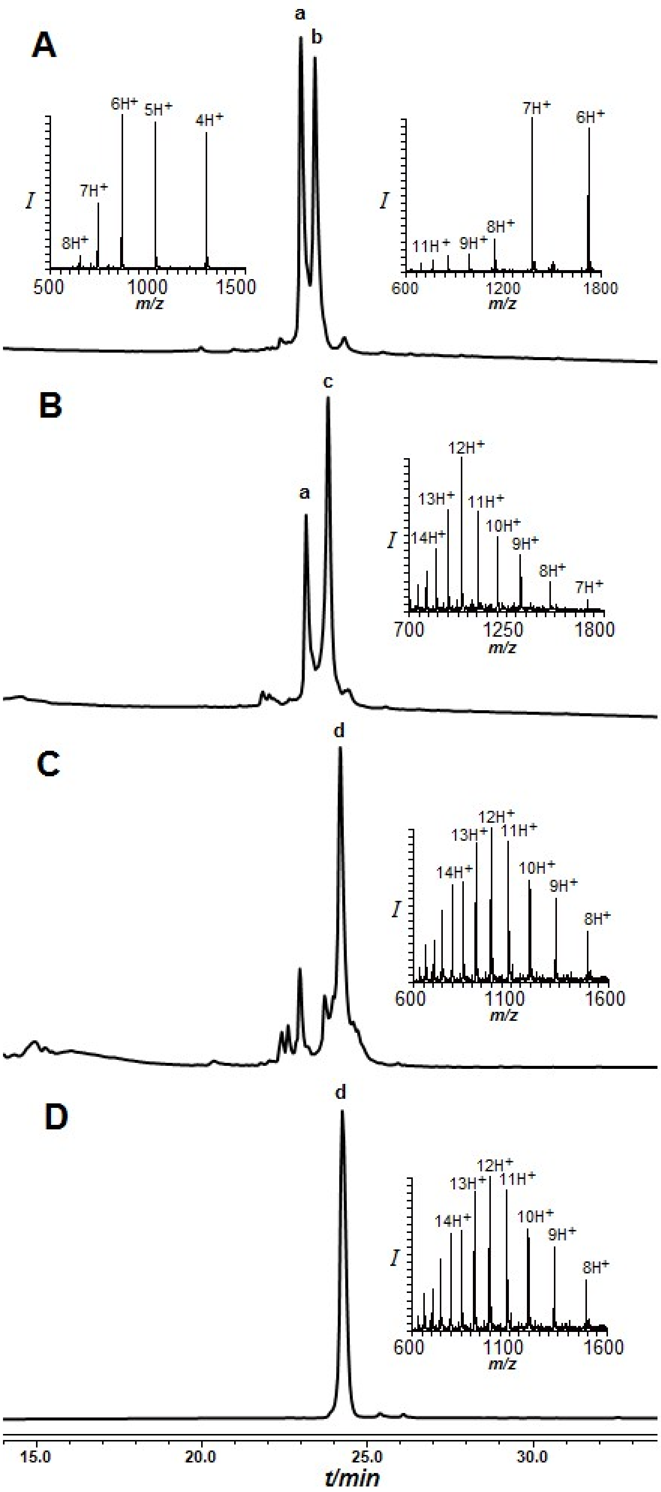

Figure S7. Synthesis of Ub ester-linked Cys-α-globin(121-150) **17**: A) Analytic HPLC and mass traces of the ligation at 0 min, peak **a** corresponds to Ub(1-45)-MMP fragment **15** and peak **b** corresponds to Cys-Ub(46-76) ester-linked Thz-α-globin(121-150) fragment **10**. B) Ligation after 1 h, peak **c** corresponds to the ligation product with observed mass 11962 Da (calcd 11962.8 Da). C) Analytical HPLC of the removal of Thz on Cys by Pd complex after 1 h (peak **d**, observed mass 11950 Da, calcd 11950.8 Da). D) Analytical HPLC and mass analysis of the purified Ub ester-linked Cys-α-globin(121-150) **17** (peak **d**, observed mass 11950 Da, calcd 11950.8 Da).

Synthesis of Ub ester-linked HA-α-globin

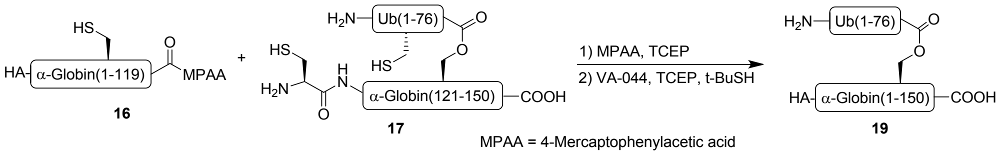

HA-α-globin(1-119)-MPAA **16** (3.0 mg, 2.3×10^{-4} mmol) and Ub ester-linked Cys-α-globin(121-150) **17** (3.3 mg, 2.76×10^{-4} mmol) were dissolved in 6 M Gn·HCl, 200 mM phosphate buffer (150μL) containing MPAA (100 mM) and TCEP (50 mM) at pH 7.2. After incubation at 37 ºC for 4 h, the reaction mixture was dialyzed using a Slide-A-Lyzer 3.5K dialysis cassette (Thermo scientific, 0.1-0.5 mL) in 6 M Gn·HCl, 200 mM phosphate buffer (500 mL) for 12 h. Then the mixture (300 uL) was subjected for the desulfurization by treating it with TCEP (23.5 mg, 0.25 mM), VA-044 (11.2 mg, 150 equiv) and t-BuSH (33 μL) 37 ºC for 6 h.[8] The progress of the reaction was monitored using analytical HPLC (C4 column) with the gradient of 0-60% B over 30 min. The product **19** was purified using a C4 semi-preparative column with the gradient of 5-55% B over 45 min solvent system to obtain 1.8 mg from the product (31 % yield).

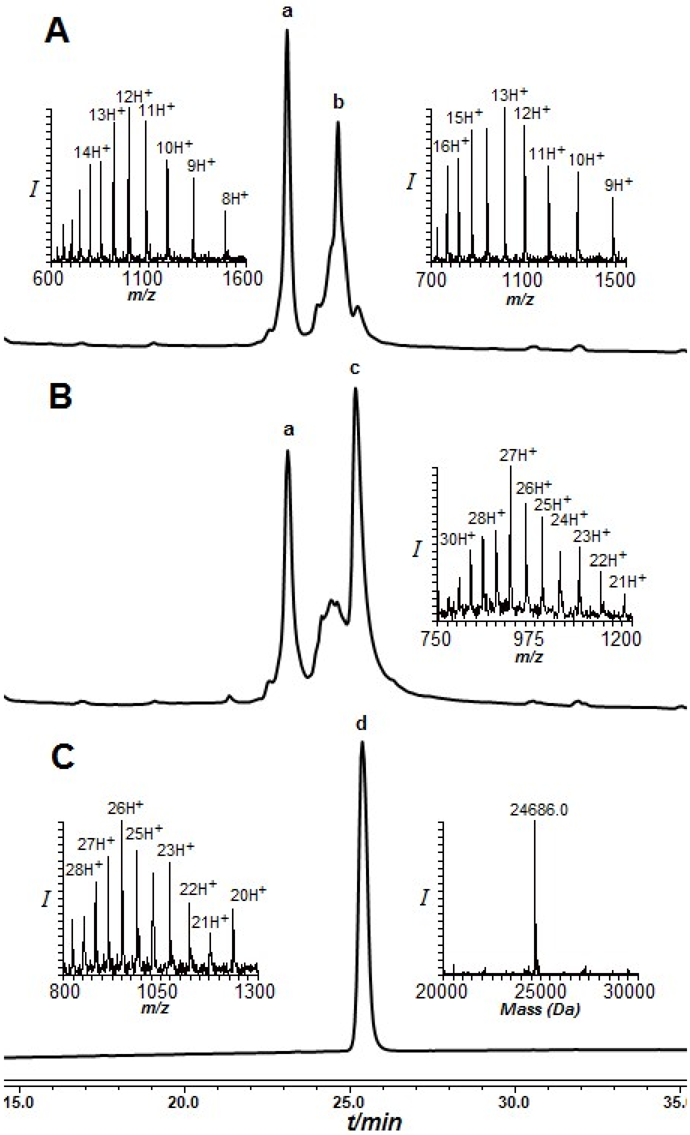

Figure S8. Synthesis of Ub ester-linked HA-α-globin **19**: A) Analytical HPLC and mass traces of the ligation at 0 min, peak **a** corresponds to Ub ester-linked Cys-α-globin(121-150) **17** and peak **b** corresponds to HA-α-globin(1-119)-MPAA **16**. B) Ligation after 1h, peak **c** corresponds to the ligation product with observed mass 24781.2 Da (calcd 24783.4 Da). C) Analytical HPLC of the purified Ub ester-linked HA-α-globin **19** after desulfurization (peak **d**, observed mass 24686 Da, calcd 24687.2 Da).

Synthesis of Ub isopeptide-linked Cys-α-globin(121-150)

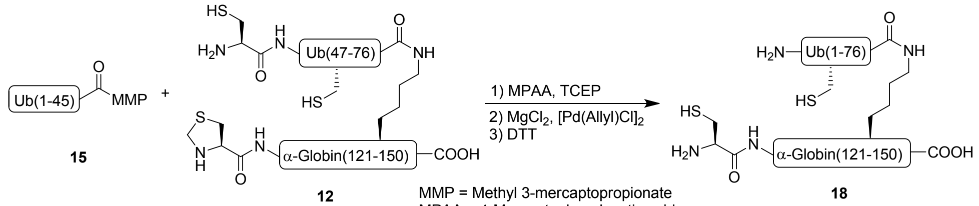

15, 12, 18

MMP = Methyl 3-mercaptopropionate
MPAA = 4-Mercaptophenylacetic acid

The ligation and Thz deprotection to the synthesis of Ub isopeptide-linked Cys-α-globin(121-150) **18** were performed similarly to the procedure described above for the ester-linked analogue **17**. Cys-Ub(47-76) isopeptide-linked Thz-α-globin(121-150) **12** (13.8 mg, 2.0×10^{-3} mmol) and Ub(1-45)-MMP **15** (12.5 mg, 2.4×10^{-3} mmol) were dissolved in 6 M Gn·HCl, 200 mM phosphate buffer (1000 μL) containing MPAA (60 mM) and TCEP (30 mM) at pH 7.2. The reaction was incubated at 37 ºC for 4 h. Then 50 equiv of $MgCl_2$ (9.5 mg) was added to the reaction mixture and the reaction was incubated at 37 ºC for 10 min. After that, the reaction mixture was treated with 15 equiv of $[Pd(Ally)Cl]_2$ (11 mg) and incubated at 37 ºC for 1 h to complete the Thz removal. The reaction was quenched by adding 60 equiv of DTT, followed by centrifugation and injection of the supernatant into the HPLC for analysis and purification. The progress of the reaction was monitored using analytical HPLC (C4 column) with the gradient of 0-60% B over 30 min. The product **18** was purified using a C4 preparative column with the gradient of 5-55% B over 45 min solvent system to obtain 9.3 mg from the product (39 % yield).

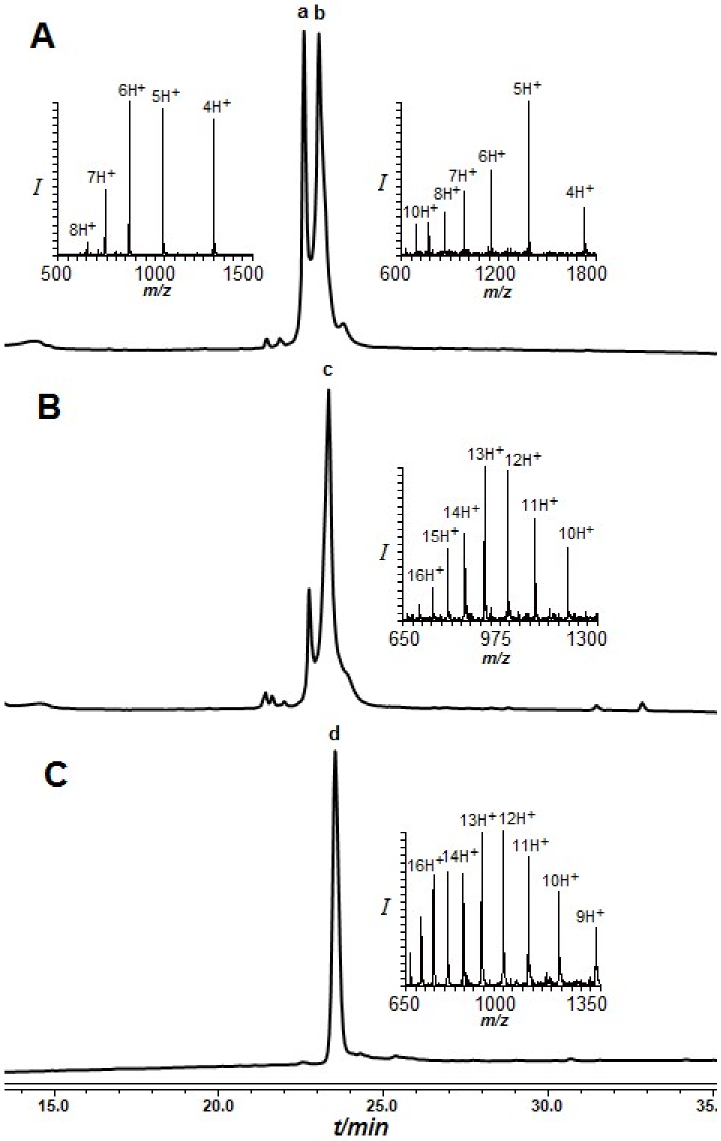

Figure S9. Synthesis of Ub isopeptide-linked Cys-α-globin(121-150) **18**: A) Analytical HPLC and mass traces of the ligation at 0 min, peak **a** corresponds to Ub(1-45)-MMP fragment **15** and peak **b** corresponds to Cys-Ub(47-76) isopeptide-linked Thz-α-globin(121-150) **12**. B) Ligation after 1 h, peak **c** corresponds to the ligation product with observed mass 11990 Da (calcd 11989.9 Da). C) Analytical HPLC and mass analysis of the purified Ub isopeptide-linked Cys-α-globin(121-150) **18** after Thz removal by Pd complex (peak **d**, observed mass 11978 Da, calcd 11977.8 Da).

Synthesis of Ub isopeptide-linked HA-α-globin

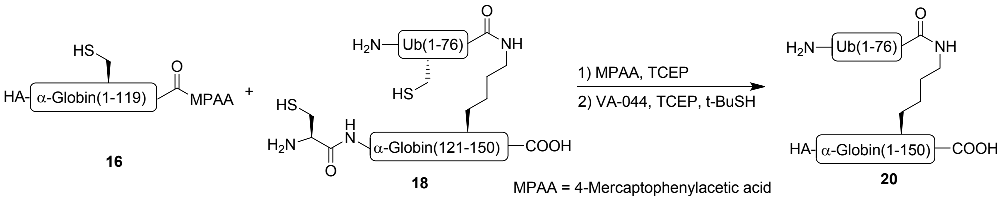

The ligation and desulfurization to the synthesis of Ub isopeptide-linked HA-α-globin **20** were performed similarly to the procedure described above for the ester-linked analogue **19**. HA-α-globin(1-119)-MPAA **16** (3.0 mg, 2.3×10^{-4} mmol) and Ub isopeptide-linked Cys-α-globin(121-150) **18** (2.75 mg, 2.3×10^{-4} mmol) were dissolved in 6 M Gn·HCl, 200 mM phosphate buffer (150 μL) containing MPAA (100 mM) and TCEP (50 mM) at pH 7.2. After it was incubated at 37 ºC for 4h, the reaction mixture was dialyzed using a Slide-A-Lyzer 3.5K dialysis cassette (Thermo scientific, 0.1-0.5 mL) in 6 M Gn·HCl, 200 mM phosphate buffer (500 mL) for 12 h. Then the mixture (300 uL) was subjected for the desulfurization by treating with TCEP (23.5 mg, 0.25 mM), VA-044 (11.2 mg, 150 equiv) and t-BuSH (33 μL) at 37 ºC for 6 h. The progress of the reaction was monitored using analytical HPLC (C4 column) with the gradient of 0-60% B over 30 min. The product **20** was purified using a C4 semi-preparative column with the gradient of 5-55% B over 45 min solvent system to obtain 1.8 mg from the product (31% yield).

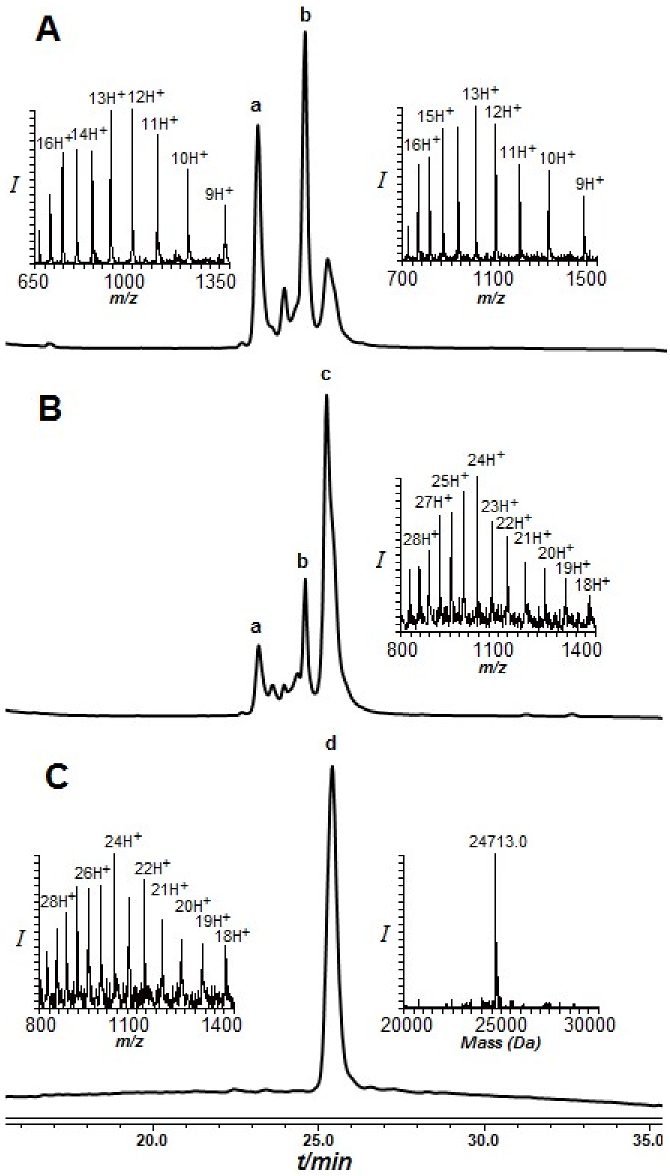

Figure S10. Synthesis of Ub isopeptide-linked HA-α-globin **20**: A) Analytical HPLC and mass traces of the ligation at 0 min, peak **a** corresponds to Ub isopeptide-linked Cys-α-globin(121-150) **18** and peak **b** corresponds to HA-α-globin(1-119)-MPAA **16**. B) Ligation after 1 h, peak **c** corresponds to the ligation product with observed mass 24810 Da (calcd 24810.5 Da). C) Analytical HPLC of the purified the Ub isopeptide-linked HA-α-globin **20** after desulfurization (peak **d**, observed mass 24713 Da, calcd 24714.3 Da).

Deubiquitination assays of Ub ester-linked HA-α-globin and wild type with different DUBs: To 20 uL Tris buffer solution (50 mM TRIS base, 1 mM TCEP, pH 7.7) containing 1 uM Ub ester-linked HA-α-globin, the deubiquitinase enzyme (50 mM Tris, 0.5 mM EDTA, 1 mM TCEP and 0.5 mg/ml ovalbumin, pH 7.5) (enzyme: substrate 1:10 ratio) was added. All the reactions were incubated at 37 ºC for 5 mins. The substrate without enzyme was also incubated for 5 mins. All the reactions were quenched with 1 M of HCl and resolved with 14% Tricine SDS-PAGE. The Gels were transferred and immunoblotted with anti-ubiquitin antibody polyclonal rabbit-anti-UbDako (z0458) in a 1:10,000 dilution. Goat pAb to Rb HRP IgG (HRP) conjugate (Biorad 170-6515) was used as the secondary antibody in a 1:20,000 dilution. For the detection of the bends, image quant LAS 4000 (GE Healthcare) was used.

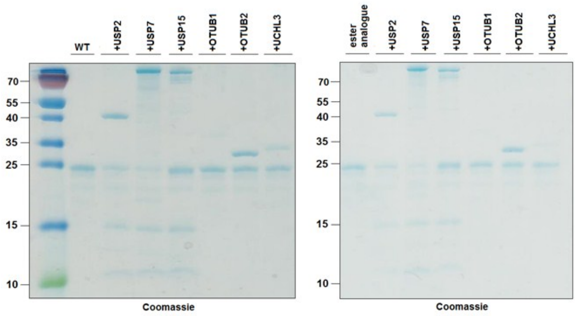

Figure S11. Coomassle analysis of ester- and isopeptide-linked HA-α-globin-Ub treated with panel of DUBs. The analogues were treated with 1:10 enzyme to substrate molar ratio.

Deubiquitination assays of Ub ester-linked HA-α-globin and wild type with USP2 and USP15: To 120 uL Tris buffer solution (50 mM Tris base, 1 mM TCEP, pH 7.7) containing 1 uM Ub ester-linked HA-α-globin, enzyme (50 mM Tris, 0.1 mM EDTA, 1 mM TCEP and 0.5 mg/ml ovalbumin, pH 7.5) (enzyme: substrate 1:50 ratio) was added. The reactions were incubated at 37 ºC. After different times, 20 uL of the reaction mixture was taken out and quenched with 1 M of HCl and resolved with 14% Tricine SDS-PAGE. The Gels were transferred and immunoblotted with anti-ubiquitin antibody polyclonal rabbit-anti-UbDako (z0458) in a 1:10,000 dilution. Goat pAb to Rb HRP IgG (HRP) conjugate (Biorad 170-6515) was used as the secondary antibody in a 1:20,000 dilution. For the detection of the bends, image quant LAS 4000 (GE Healthcare) was used.

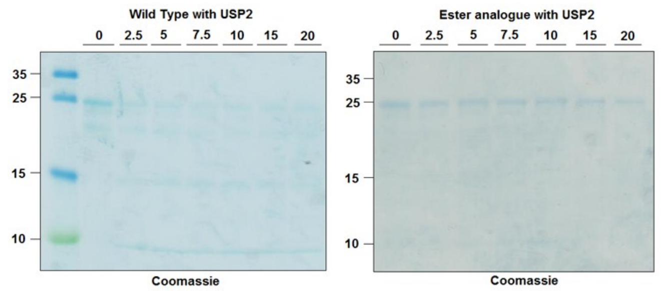

Figure S12. Coomassle analysis of ester-linked HA-α-globin-Ub (right gel) and wild type (left gel). The analogues were treated with USP2 at 1:50 (enzyme: substrate) molar ratio for different time points. Untreated analogues were used as a control.

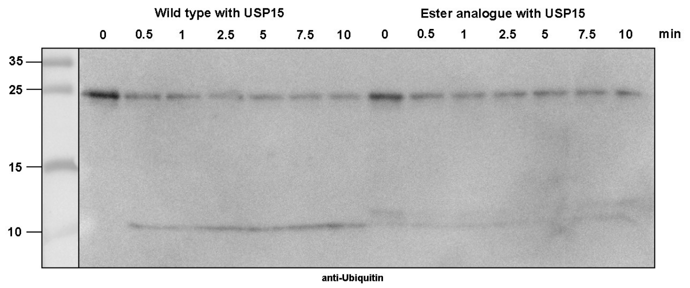

Figure S13. Western blot analysis of ester-linked HA-α-globin-Ub (right gel) and wild type (left gel). The analogues were treated with USP15 at 1:50 (enzyme: substrate) molar ratio for different time point. Untreated analogues were used as a control.

References

1. J. B. Blanco-Canosa, B. Nardone, F. Albericio and P. E. Dawson, *J. Am. Chem. Soc.*, 2015, **137**, 7197-7209.

2. J. B. Blanco-Canosa and P. E. Dawson, *Angew. Chem. Int. Ed. Engl.*, 2008, **47**, 6851-6855.

3. L. J. Cruz, N. G. Beteta, A. Ewenson and F. Albericio, *Org. Process. Res. Dev.*, 2004, **8**, 920-924.

4. L. A. Erlich, K. S. Kumar, M. Haj-Yahya, P. E. Dawson and A. Brik, *Org. Biomol. Chem.*, 2010, **8**, 2392-2396.

5. M. Jbara, S. K. Maity, M. Seenaiah and A. Brik, *J. Am. Chem. Soc.*, 2016, **138**, 5069-5075.

6. M. Jbara, S. K. Maity and A. Brik, *Angew. Chem. Int. Ed. Engl.*, 2017, **56**,10644 –10655.

7. J. S. Zheng, S. Tang, Y. K. Qi, Z. P. Wang and L. Liu, *Nature Protocols*, 2013, **8**, 2483-2495.

8. T. Moyal, H. P. Hemantha, P. Siman, M. Refua and A. Brik, *Chem. Sci.*, 2013, **4**, 2496-2501.
